# Editorial: Microbiome and microbial informatics

**DOI:** 10.3389/fmicb.2022.1054811

**Published:** 2022-10-19

**Authors:** Pengfei Ding, Zhenhua Ming, Juntao Liu, Ivan Erill, Zheng Zhang

**Affiliations:** ^1^Department of Chemistry and Biochemistry, University of Maryland Baltimore County, Baltimore, MD, United States; ^2^State Key Laboratory for Conservation and Utilization of Subtropical Agro-Bioresources, College of Life Science and Technology, Guangxi University, Nanning, China; ^3^School of Mathematics and Statistics, Shandong University, Weihai, China; ^4^Department of Biological Sciences, University of Maryland Baltimore County, Baltimore, MD, United States; ^5^State Key Laboratory of Microbial Technology, Institute of Microbial Technology, Shandong University, Qingdao, China

**Keywords:** microbiome, bioinformatics, evolutionary and genomic microbiology, metagenomics, data mining, computational techniques

The advancement of genome sequencing technologies and metagenomic analysis has allowed researchers to study microorganisms, as well as their functions and microbial-based interactions in natural and industrial environments. Nevertheless, the large amounts of information resulting from these studies must be stored, structured, indexed, analyzed, and correlated with existing experimental data. The requirement has led to the exploitation of bioinformatics solutions at the cross-over point of information science and microbiology.

In this context, we are pleased to note that the Microbiome and Microbial Informatics Research Topic has drawn the contributions of lots of well-respected researchers in the field all over the world, including those from China, India, Germany, the Netherlands, Saudi Arabia and Australia. We received 28 submissions, 17 of which were accepted for publication after peer review. These publications focused on new insights, novel developments, current challenges, and future perspectives in the field of microbiome and microbial informatics. We sincerely thank all researchers who have agreed to contribute to our Research Topic.

Profiting from the current rapid progress of artificial intelligence techniques, the aggregation of statistical analysis methodologies and predictions for large-scale data has evolved for a variety of fields associated with data science. Jiang et al. summarized the application and advancement on machine learning and deep learning in microbiology. They illustrated and contrasted the benefits and drawbacks of distinct algorithmic tools in four dimensions: microbiome and taxonomy, microbial ecology, pathogen and epidemiology, and drug discovery, demonstrating the development prospects of computational microbiology from the perspective of machine learning. As an example, by combining data augmentation techniques, López et al. utilized machine learning methods to investigate the predictability of smoking habits from class imbalanced saliva microbiome data to account for class imbalance. In doing so, they successfully addressed the class imbalance problem in microbiome data, resulting in a reliable prediction of smoking habits.

The rapid development of high-throughput, culture-independent analytical techniques has brought a wealth of experimental data that have significantly facilitated the human microbiome study. To study the explicit microbial variance in the human face, Wei et al. reassessed data from 822 shotgun-metagenomic sequencing of Han Chinese individuals in conjunction with 97 North American samples from the NIH Human Microbiome Project (HMP). This study explores the fine-scale facial location-related variations of skin microbiomes to provide an in-depth understanding of ecological processes that underlie facial microbial changes. Wang et al. analyzed 404 datasets from human oral saliva samples and made comparisons with other human part samples to reveal the diversity and biogeography of human oral saliva microbial communities. Using high-throughput sequencing of 16S rRNA V3-V4 hypervariable regions, Huang, Deng et al. assessed the fecal microbiota profiles of healthy individuals from three representative Han populations in Guangdong Province, China. On the basis of genus-level OTU abundance, the random forest prediction model indicated that there may be potential to distinguish individuals according to their fecal microbial community. Li et al. analyzed the evolution of the gut microbiome in Tibetan populations in the Minjiang River Basin. This study demonstrates that altitude of habitation is a vital factor influencing the enterotype of the Tibetan population microbiome. Sindi et al. concluded that short-term maternal dietary interventions during lactation could significantly alter the functional potential of the gut microbiome of breastfed infants. Another study by Peng et al. showed that esterases from *Bifidobacteria* undertake albiflorin conversion in the gut and play an important role in the metabolism of natural compounds including ester bonds. *Bifidobacteria*-mediated metabolism of ester bonds has the potential to facilitate the exploitation of novel enzymes and probiotic adjuvant compounds for therapeutic use.

The development of omics technologies has greatly increased our understanding of the interaction between microbes and agricultural animals and plants. Na et al. evaluated the effects of adding six common commercial lactic acid bacterial additives in the microbial communities and condition of fermentation of alfalfa silage. The study demonstrated that lactic acid bacterial additives enhanced the quality of fermentation and changed the microbial communities of alfalfa silage. Zhang et al. investigated the reaction in fungal subcommunities in a corn-wheat rotation plow land managed by long-term conservation tillage. Their findings indicated that the use of no-tillage and straw mulching practices had a negative impact on the complicacy of plentiful and medium fungal networks, but did not prominently affect rare fungal networks. Their study informs our learning on the reaction in fungal subcommunities to preservation tillage management technologies, and provides a new view on how fungal subcommunity assemble. Wen et al. reported the discovery of a new NAD(P)-dependent alcohol dehydrogenase from *Gluconobacter frateurii* NBRC 3264, which displayed great potentiality for application in processes involving high-yield bioconversion of D-allulose and could therefore be used for the manufacturing production of D-allulose.

Molecular diagnostics are extensively applied in clinical microbiology studies, such as routine detection and epidemiological analysis of infectious microbes. Liao et al. presented a concise multilocus sequence typing protocol for *Staphylococcus aureus* and demonstrated the effectiveness of portable sequencing technology for accurate, rapid, and routine molecular typing.

Molecular taxonomy and environmental adaptation have been deeply studied due to the increased genomic information of some microbial species. For example, Du et al. isolated a novel pathogenic bacterium, *Haemobacillus shengwangii*, from a blood sample of a critically ill patient. They classified *H. shengwangii* as a member of the Thermicanaceae family, for which they report the first high-quality genome, by utilizing single-molecule real-time sequencing and next-generation sequencing technologies. Mahata et al. combined morphological descriptions, phylogenetics and single-nucleotide polymorphism analysis to characterize the distribution and relative abundance of *Aspergillus* species from *Foeniculum vulgare*. The integration of morphological features with molecular systematics is regarded as an essential element of taxonomic studies. Huang, Peng et al. isolated and identified 22 fungal strains from the Beibu Gulf coral using serial dilution and internal transcribed spacer sequence analysis. The isolates were further divided into three branches by phylogenetic analysis. Their study provided eight fungal isolates with potential activity against *Vibrio* species, and two alkaloid-type antibiotics with anti-Vibrio effects were characterized from the bioactive strain *Fusarium equiseti* BBG10. Liu et al. characterized the diversity and function of laccase family genes in the fungus *Schizophyllum commune* 20R-7-F01 genome, which was isolated from deep sea sediment. Their findings contribute to further our understanding of laccase genes in white-rot fungi and pave the way for further exploring the relationship between the laccase gene family and anaerobic degradation of lignin by *Schizophyllum commune*. Yuan et al. conducted comparative genomic and functional analyses of *Paenibacillus peoriae* ZBSF16, a species with potential for biocontrol against grapevine diseases. Their analysis provided insight into the plant growth-promoting and biocontrol mechanisms of this bacterium, identifying conserved genes involved in both plant-growth promotion and antibiotic production.

Modern microbiology studies lead to increased adoption of high-throughput techniques and big data methods to provide faster, unbiased and more reproducible results than traditional studies with insufficient data or time-consuming pure experimental techniques. We created this Research Topic with the hope that the contributions submitted to it would prove useful for a wide audience, but in particular to microbiologists, computational biologists and bioinformaticians. We believe that the high-quality contributions published within this Research Topic, together with the diversity of microorganisms and environments studied and the broad array of experimental and computational techniques used, have amply achieved our goal.

## Author contributions

All authors listed have made a substantial, direct, and intellectual contribution to the work and approved it for publication.

## Conflict of interest

The authors declare that the research was conducted in the absence of any commercial or financial relationships that could be construed as a potential conflict of interest.

## Publisher's note

All claims expressed in this article are solely those of the authors and do not necessarily represent those of their affiliated organizations, or those of the publisher, the editors and the reviewers. Any product that may be evaluated in this article, or claim that may be made by its manufacturer, is not guaranteed or endorsed by the publisher.

